# Supplementary material for: Identification of the Notch ligand DLK1 as an immunotherapeutic target and regulator of tumor cell plasticity and chemoresistance in adrenocortical carcinoma
Source: Nat Commun. 2025 Jul 1;16:5511. doi: 10.1038/s41467-025-60649-w (PMC12216638; doi:10.1038/s41467-025-60649-w)
Supplement: Supplementary file 1 — Supplementary Information [file 41467_2025_60649_MOESM1_ESM.pdf]

Supplementary Figure 1

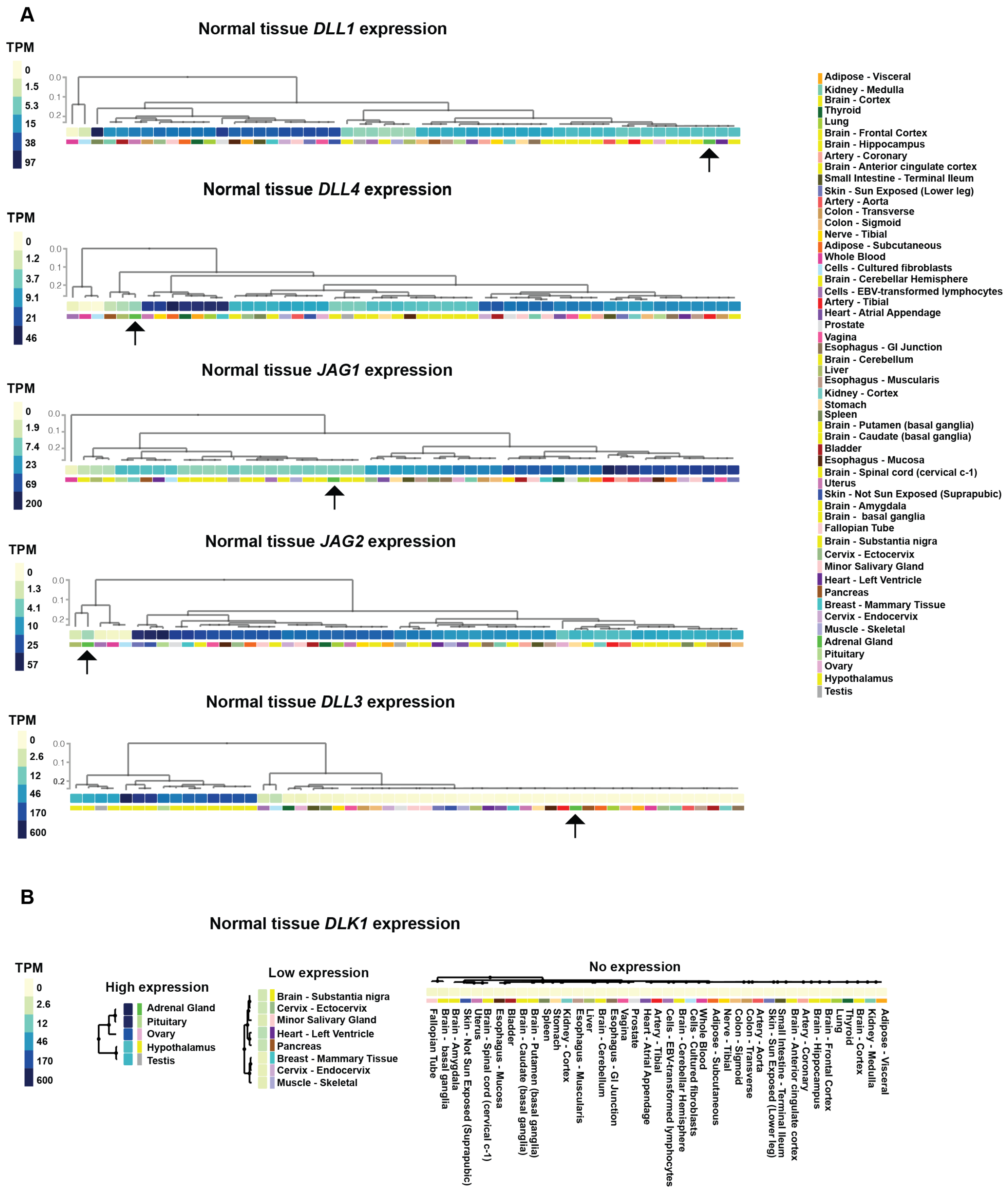

Supplementary Figure 1. Normal tissue expression of Notch ligands. (A) Normal tissue expression of *DLL1*, *DLL4*, *JAG1*, *JAG2*, and *DLL3* in the GTEx database. Arrows points to expression of each ligand in the adrenal gland. (**B**) Normal tissue expression of *DLK1* in the GTEx database**.** Source data are provided as a Source Data file.

Supplementary Figure 2

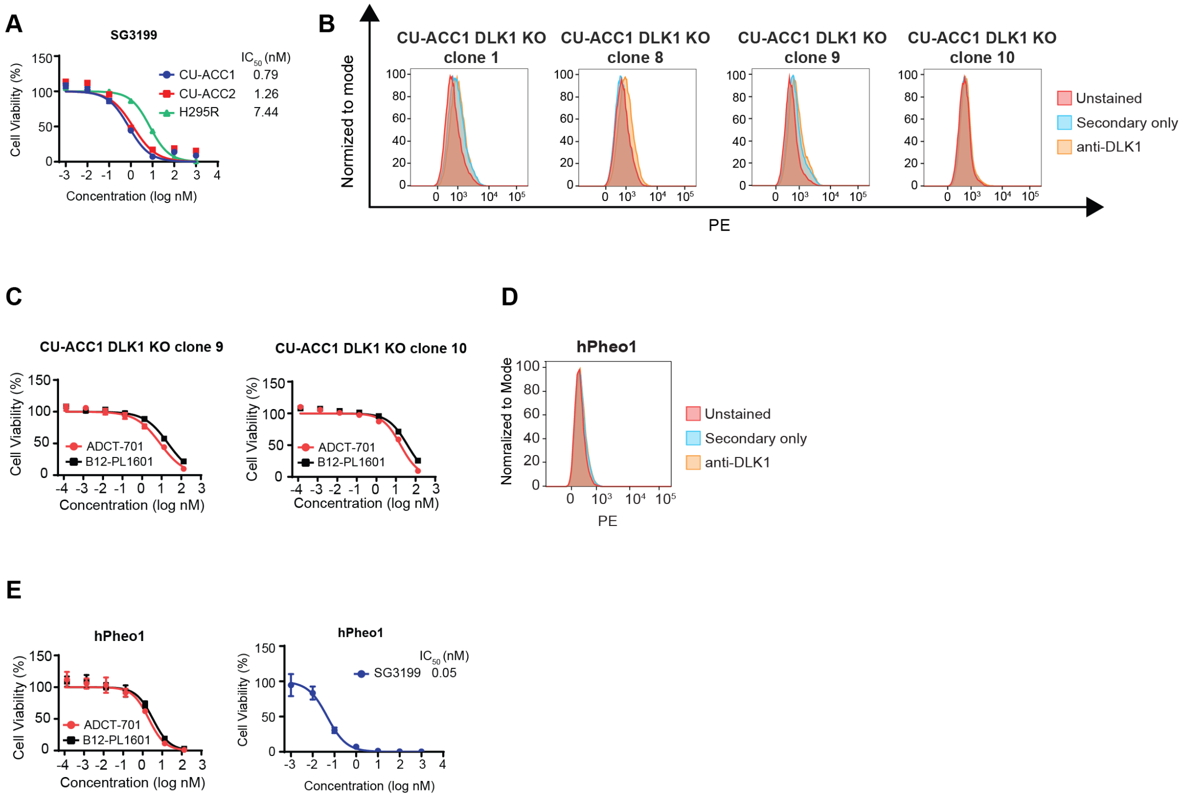

Supplementary Figure 2. ADCT-701 cytotoxicity is DLK1 dependent. (A) SG3199 cytotoxicity in CU-ACC1, CU-ACC2, and H295R cells. Cells were treated with SG3199 for 3 days (data representative of n=3 independent experiments). **(B)** Cell surface expression of DLK1 in four CU-ACC1 DLK1 KO clones (data representative of n=2 independent experiments). **(C)** Cytotoxic activity of ADCT-701 in two CU-ACC1 DLK1 KO clones. Cells were treated with ADCT-701 for 7 days (data representative of n=4 independent experiments). **(D)** Cell surface expression of DLK1 by flow cytometry in hPheo1 cell line (data representative of n=2 independent experiments). **(E)** Cytotoxic activity of ADCT-701 and SG3199 in hPheo1 cell line. Cells were treated with ADCT-701 or SG3199 for 7 or 3 days, respectively (data representative of n=4 independent experiments). Error bars represent S.E.M. Source data are provided as a Source Data file.

Supplementary Figure 3

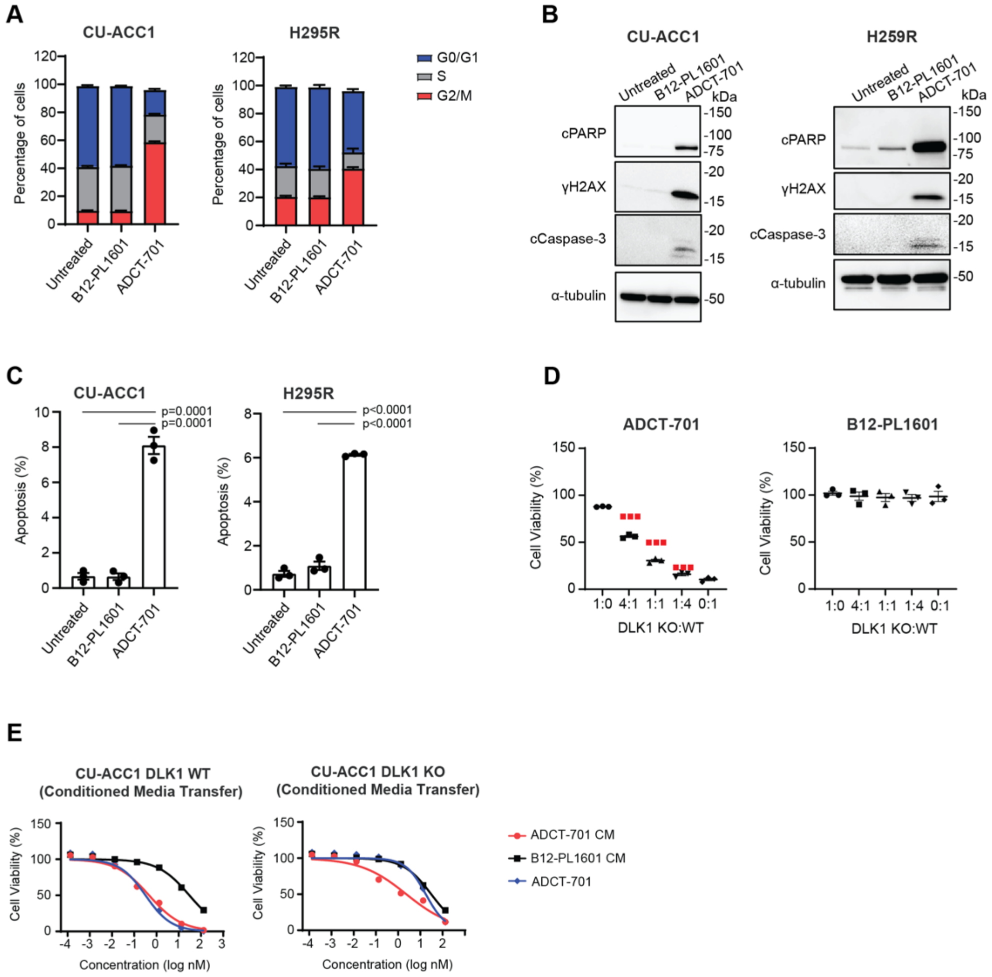

Supplementary Figure 3. ADCT-701 induces cytotoxicity in DLK1^{+} ACC cell lines through apoptosis and bystander killing. (**A**) Quantification of the cell cycle in CU-ACC1 and H295R cells treated with 0.02 µg/mL ADCT-701 or B12-PL1601 for 2 or 5 days, respectively. Data are expressed as mean ± S.E.M. (n=3 biological replicates). (**B**) Immunoblots showing DNA damage (γH2AX) and apoptosis (cleaved PARP and cleaved caspase-3) in CU-ACC1 and H295R cells treated with 20 µg/mL ADCT-701 or B12-PL1601 for 1 or 3 days, respectively. For immunoblot analysis, experiments were performed two times with similar results. (**C**) Annexin V apoptosis assay of CU-ACC1 or H295R cells treated with 20 µg/mL ADCT-701 or B12-PL1601 after 1 or 3 days of treatment, respectively. Results represent mean ± S.E.M. (n=3 biological replicates). Unpaired t tests were used to calculate two-tailed p-values. (**D**) Cell viability among different co-culture ratios of CU-ACC1 DLK1 KO:CU-ACC1 treated with 1 µg/mL of ADCT-701 (left) or B12-PL1601 (right) for 4 days. Red lines indicate expected viability with no bystander killing effect. Data are presented as the mean ± S.E.M. (n=3 independent experiments). (**E**) CU-ACC1 cells were treated with ADCT-701 or B12-PL1601 for 5 days. Conditioned media was collected and transferred to either CU-ACC1 or CU-ACC1 DLK1 KO cells for 5 days and cell viability was measured. Results represent mean ± S.E.M. from 3 independent experiments. Source data are provided as a Source Data file.

Supplementary Figure 4

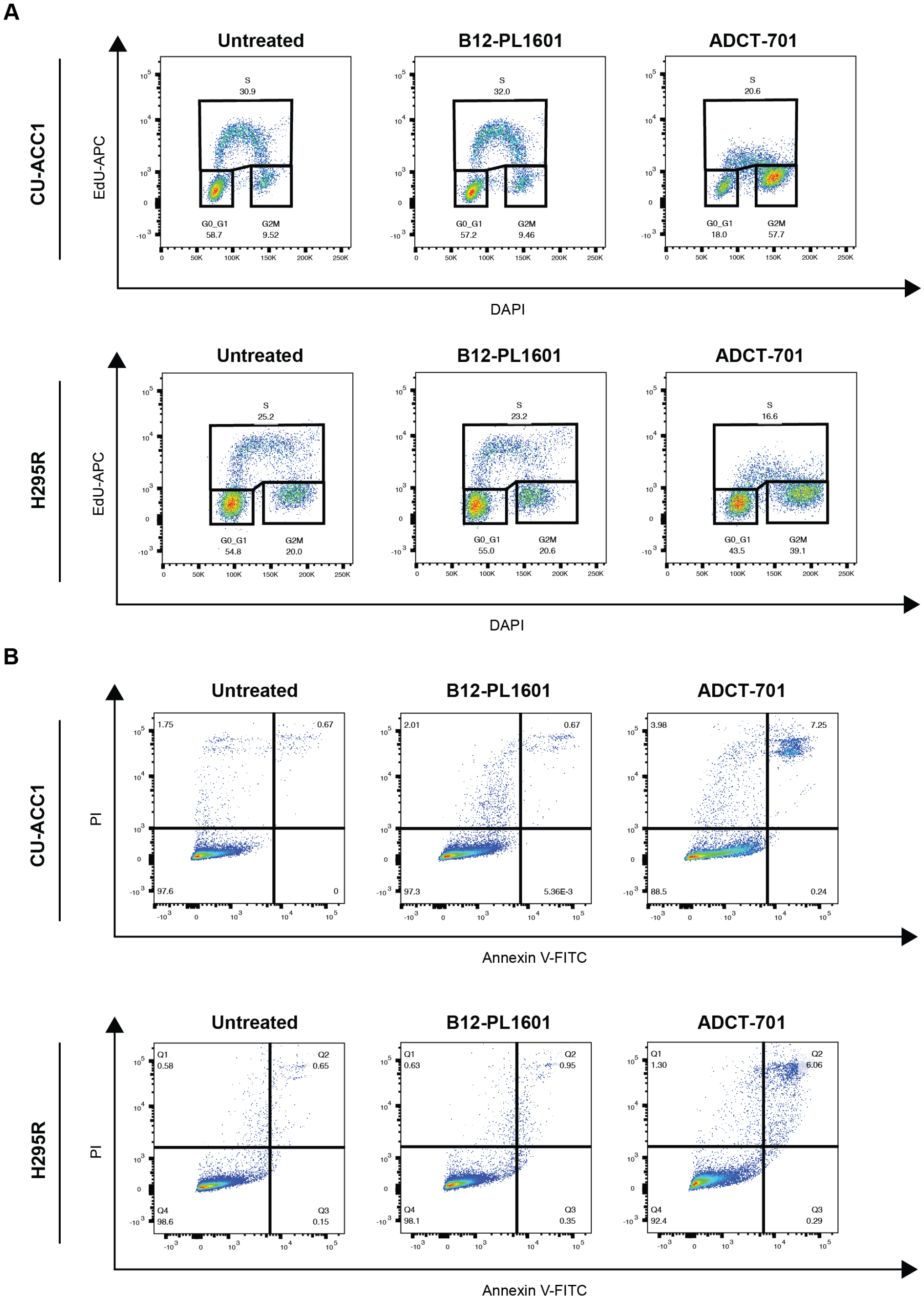

Supplementary Figure 4. ADCT-701 induces cell cycle arrest and apoptosis among DLK1$^+$ ACC cell lines. (A) Representative flow graphs of cell cycle analysis show cells in S-phase (EdU+) and other phases (G1, and G2M) in CU-ACC1 or H295R cells treated with 0.02 µg/mL ADCT-701 or B12-PL1601 for 2 or 5 days respectively. **(B)** Assessment of apoptosis by flow cytometry of Annexin V and PI double positive CU-ACC1 or H295R cells treated with 20 µg/mL ADCT-701 or B12-PL1601 for 1 or 3 days, respectively. Source data are provided as a Source Data file.

Supplementary Figure 5

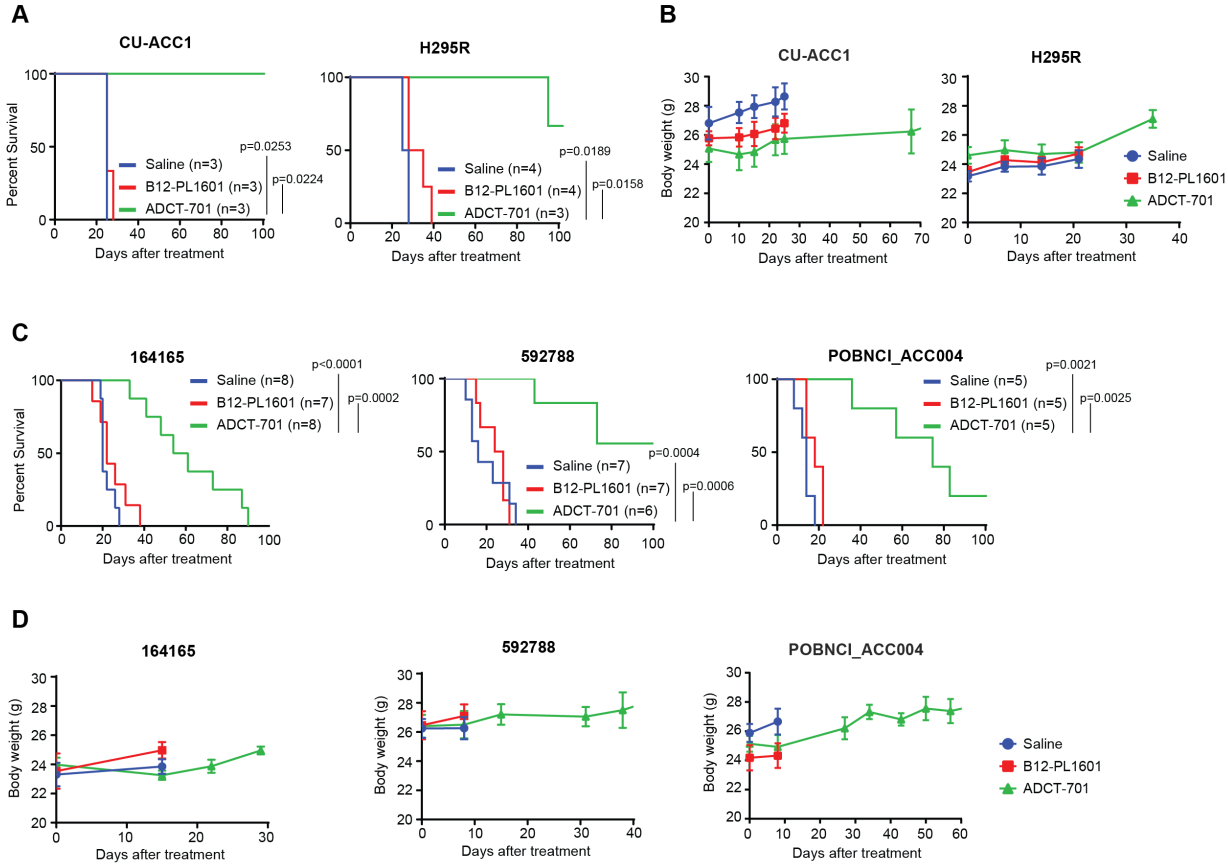

Supplementary Figure 5. ADCT-701 survival and toxicity in ACC cell-line and patient-derived xenografts. (A) CU-ACC1 and H295R xenograft survival in mice treated with normal saline (CU-ACC1: n=3; H295R: n=4), B12-PL1601 (CU-ACC1: n=3; H295R: n=4), or ADCT-701 (CU-ACC1: n=3; H295R: n=3) (1 mg/kg). **(B)** Body weight curves with saline (CU-ACC1: n=3; H295R: n=4), B12-PL1601 (CU-ACC1: n=3; H295R: n=4), or ADCT-701 (CU-ACC1: n=3; H295R: n=3) treatment (1 mg/kg) in CU-ACC1 and H295R xenografts. (**C**) ACC PDXs 164165, 592788, and POBNCI_ACC004 survival after treatment (1 mg/kg) with saline (164165: n=8; 592788: n=7; POBNCI_ACC004: n=5), B12-PL1601 (164165: n=7; 592788: n=7; POBNCI_ACC004: n=5) or ADCT-701 (164165: n=8; 592788: n=6; POBNCI_ACC004: n=5). **(D)** Body weight curves with saline (164165: n=8; 592788: n=7; POBNCI_ACC004: n=5), B12-PL1601 (164165: n=7; 592788: n=7; POBNCI_ACC004: n=5), or ADCT-701 (164165: n=8; 592788: n=6; POBNCI_ACC004: n=5) treatment (1 mg/kg) in ACC PDX models. For survival analysis, log-rank tests were used to calculate p-values. Source data are provided as a Source Data file.

Supplementary Figure 6

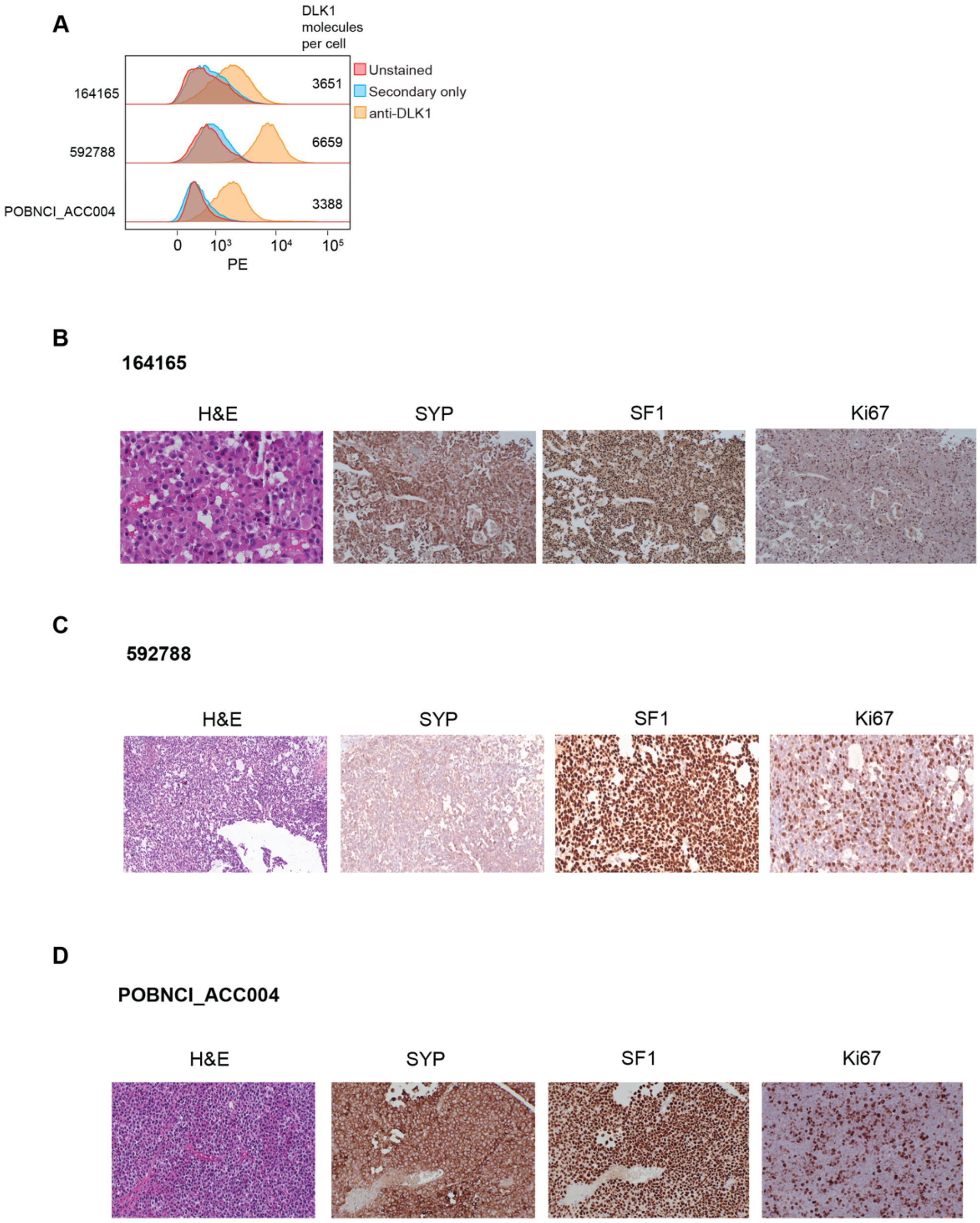

Supplementary Figure 6. DLK1 flow cytometry and validation of ACC patient-derived xenograft models. (A) Cell surface expression of DLK1 and the number of DLK1 molecules per cell among 3 ACC PDX models (data representative of n=2 independent experiments). H&E staining and immunohistochemistry for NE marker (SYP), adrenal specific marker (SF1) and proliferation marker (Ki67) in **(B)** 164165 PDX, **(C)** 592788 PDX, and **(D)** POBNCI_ACC004 PDX. Source data are provided as a Source Data file.

Supplementary Figure 7

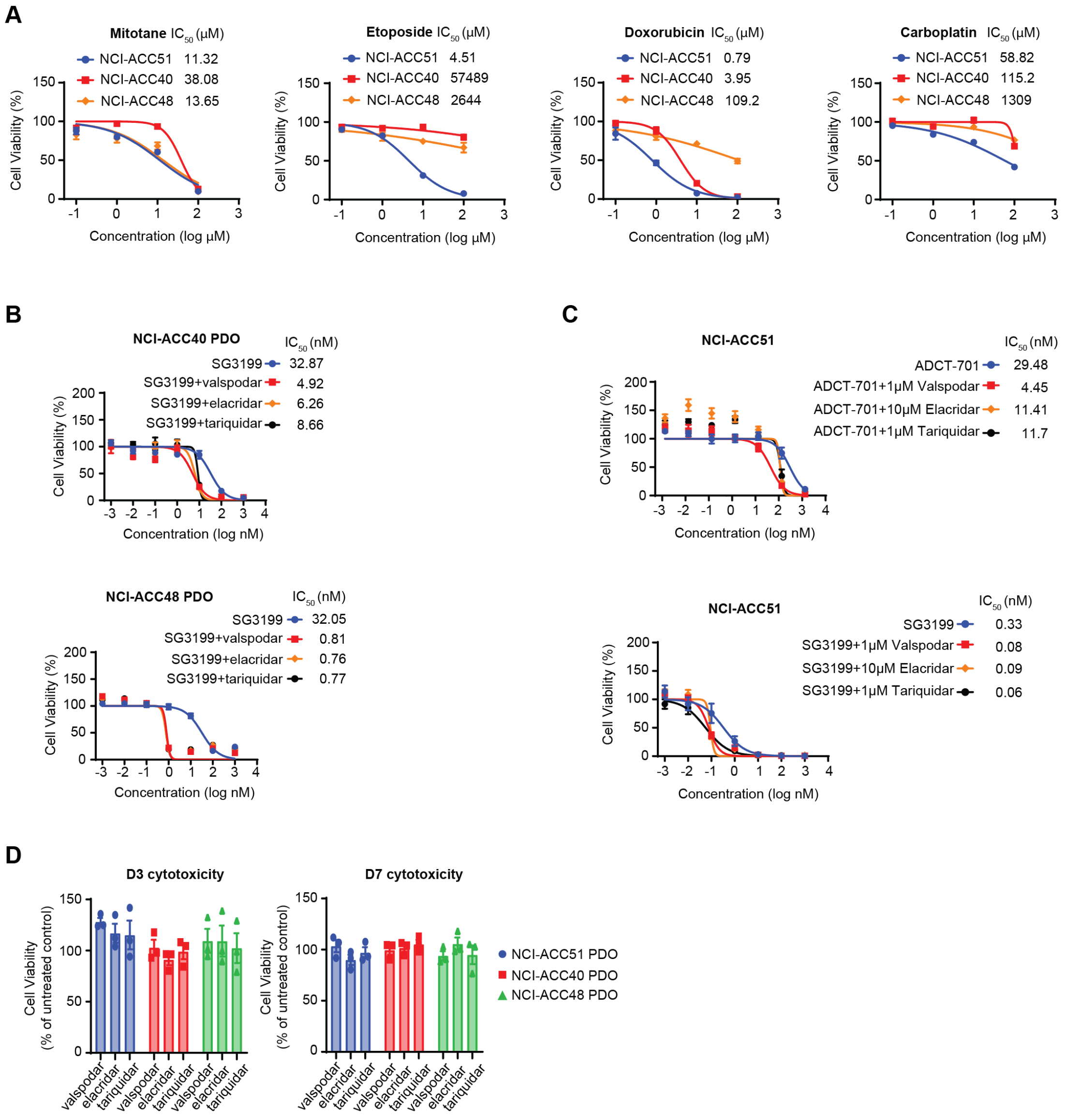

Supplementary Figure 7. ABCB1 regulates ADCT-701 and chemotherapy resistance among ACC patient-derived organoids. (A) Cytotoxic activity of mitotane, etoposide, doxorubicin, and carboplatin in three ACC patient-derived organoids (PDOs) (NCI-ACC40, NCI-ACC48, and NCI-ACC51). Cells were treated with chemotherapeutic agents as shown for 3 days (data representative of n=3 independent experiments). **(B)** SG3199 cytotoxicity in the NCI-ACC40 and NCI-ACC48 PDOs with or without ABCB1 inhibitors. Cells were treated with SG3199 combined with or without ABCB1 inhibitors for 3 days (data representative of n=3 independent experiments). **(C)** ADCT-701 and SG3199 cytotoxicity in the NCI-ACC51 PDO with or without ABCB1 inhibitors. Cells were treated with ADCT-701 (data representative of n=3 independent experiments) or SG3199 (data representative of n=3 independent experiments) combined with or without ABCB1 inhibitors for 7 or 3 days, respectively. **(D)** ABCB1 inhibitor cytotoxicity in NCI-ACC40, NCI-ACC48, and NCI-ACC51 PDOs. Cells were treated with each inhibitor for 3 or 7 days (n=3 independent experiments). Error bars represent S.E.M. Source data are provided as a Source Data file.

Supplementary Figure 8

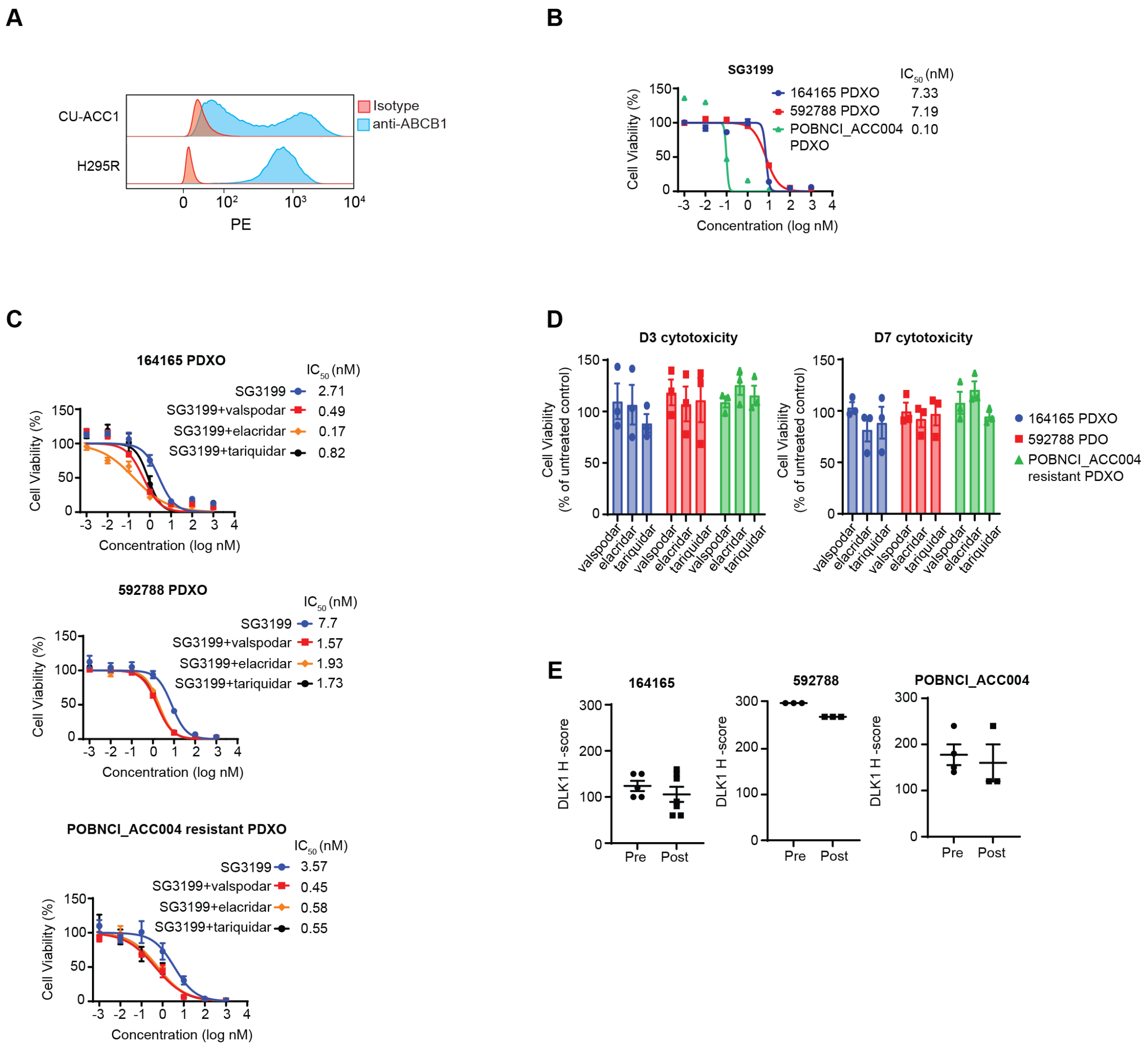

Supplementary Figure 8. ADCT-701 cytotoxicity in ACC PDX-derived organoids with or without ABCB1 inhibitors. (A) Flow cytometry histograms of ABCB1 in CU-ACC1 and H295R cell lines. Shaded gray histograms represent isotype controls for each condition (data representative of n=2 independent experiments). **(B)** SG3199 *in vitro* cytotoxicity in 164165, 592788 and POBNCI_ACC004 PDX-derived organoids. **(C)** SG3199 cytotoxicity in 164165, 592788 and resistant POBNCI_ACC004 PDX-derived organoids treated with or without ABCB1 inhibitors. For panels **B** and **C**, cells were treated with SG3199 or SG3199 combined with or without ABCB1 inhibitors for 3 days, respectively (data representative of n=3 independent experiments). **(D)** ABCB1 inhibitor cytotoxicity in 164165, 592788 and resistant POBNCI_ACC004 PDX-derived organoids. Cells were treated with each inhibitor for 3 or 7 days (n=3 independent experiments). **(E)** DLK1 immunohistochemistry in PDX tumors prior to ADCT-701 treatment (Pre) and after resistance to ADCT-701 (Post). Error bars represent S.E.M. Source data are provided as a Source Data file.

Supplementary Figure 9

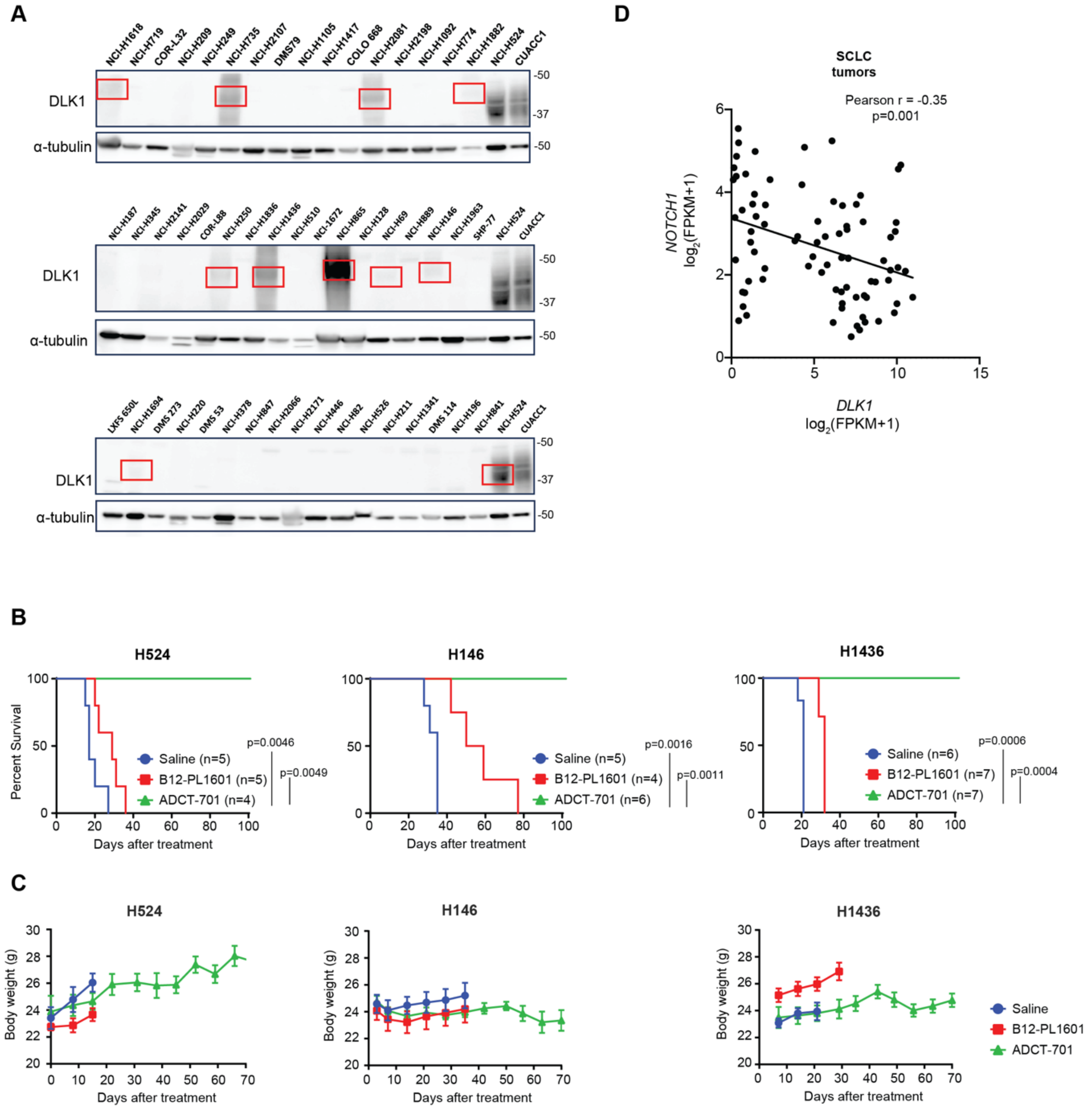

Supplementary Figure 9. Survival and toxicity of ADCT-701 treatment in DLK1⁺ SCLC xenograft models. (A) Immunoblot analysis of DLK1 across 51 SCLC cell lines. Cell lines with positive expression of DLK1 are demarcated by the red rectangles. **(B)** Survival curves of 3 SCLC xenograft models after treatment (1 mg/kg) with saline (H524: n=5; H146: n=5; H1436: n=6), B12-PL1601 (H524: n=5; H146: n=4; H1436: n=7) or ADCT-701 (H524: n=4; H146: n=6; H1436: n=7). Log-rank tests were used to calculate p-values. **(C)** Body weight curves with saline (H524: n=5; H146: n=5; H1436: n=6), B12-PL1601 (H524: n=5; H146: n=4; H1436: n=7), or ADCT-701 (H524: n=4; H146: n=6; H1436: n=7) treatment (1 mg/kg) in SCLC xenografts. **(D)** Correlation between *NOTCH1* and *DLK1* expression among SCLC primary tumors. Source data are provided as a Source Data file.

Supplementary Figure 10

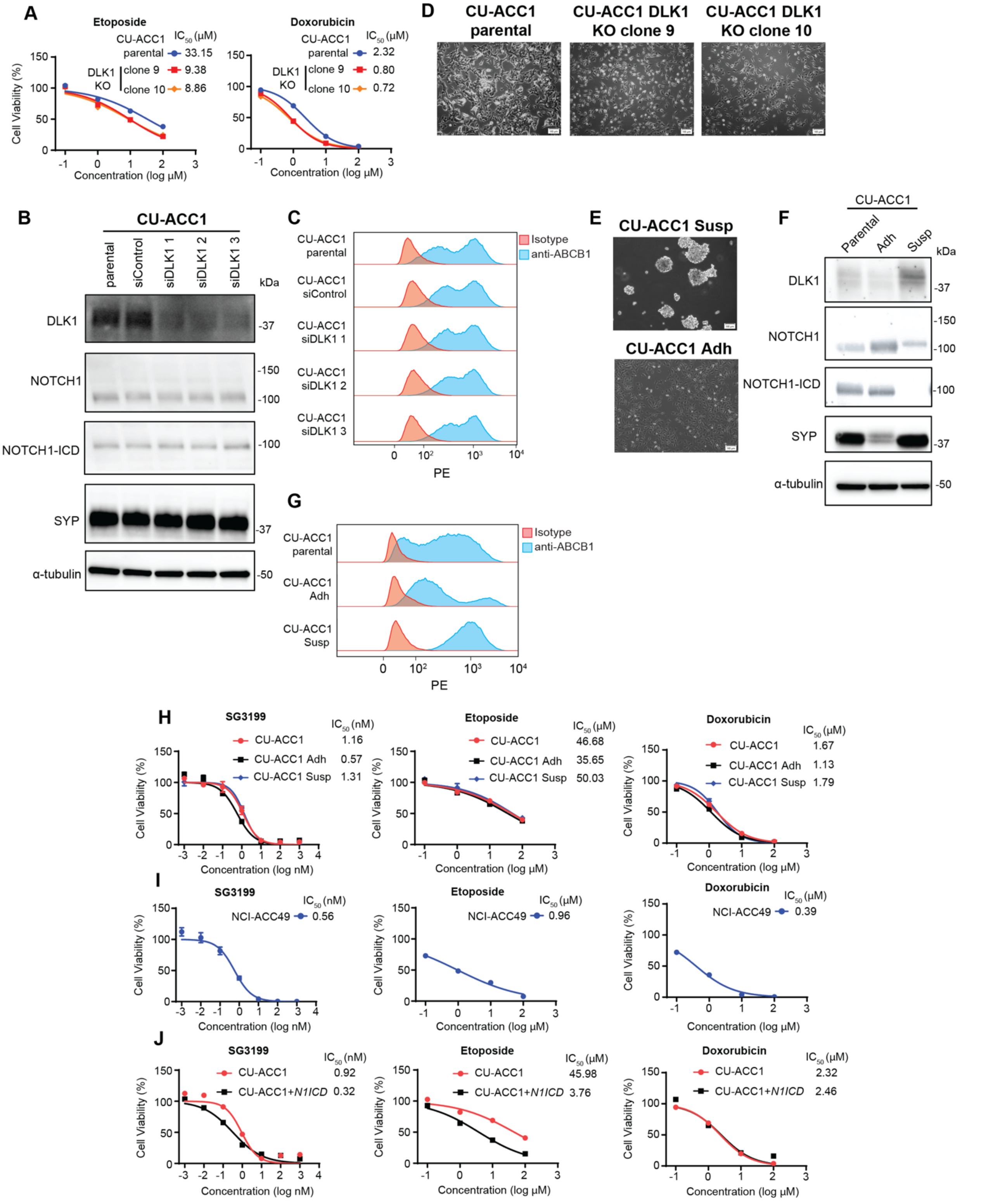

Supplementary Figure 10. Additional data supporting the relationship between DLK1, NOTCH1 signaling, ABCB1, and chemoresistance in ACC. (A) Cytotoxic activity of etoposide and doxorubicin in CU-ACC1 parental and DLK KO clones 9 and 10 cells. **(B)** Immunoblot analysis of DLK1, total NOTCH1 and NOTCH1 intracellular domain (ICD), NE marker synaptophysin (SYP), and loading control (α-tubulin) proteins in siControl compared to siDLK1 in CU-ACC1 cells. **(C)** Flow cytometry histograms of ABCB1 expression in CU-ACC1 cells with and without short-term DLK1 knockdown. **(D)** Photomicrographs of CU-ACC1 parental and DLK KO clones 9 and 10 cells. **(E)** Photomicrographs of CU-ACC1 suspension and adherent cells. **(F)** Immunoblot analysis of DLK1, total NOTCH1 and NOTCH1-ICD, SYP, and α-tubulin proteins in CU-ACC1 parental, adherent, and suspension cells. **(G)** Flow cytometry histograms of ABCB1 expression in CU-ACC1 parental, adherent, and suspension cells. Cytotoxic activity of SG3199, etoposide, and doxorubicin in **(H)** CU-ACC1 parental, adherent, and suspension cells, **(I)** patient-derived organoid NCI-ACC49, and **(J)** CU-ACC1 cells with and without *N1ICD* overexpression. For panels **A**, **H**, **I**, and **J**, cells were treated with SG3199 or other chemotherapeutic drugs for 3 days. For cell line cytotoxicity, data are representative of n=3 independent experiments. SG3199 or other chemotherapeutic drugs cytotoxicity data in NCI-ACC49 PDO are representative of n=3 independent experiments. For immunoblot analysis, experiments were performed two times with similar results. Scale bars represent 100 μm. Error bars represent S.E.M. Shaded gray histograms represent isotype controls for each condition (data representative of n=2 independent experiments). Source data are provided as a Source Data file.

Supplementary Figure 11

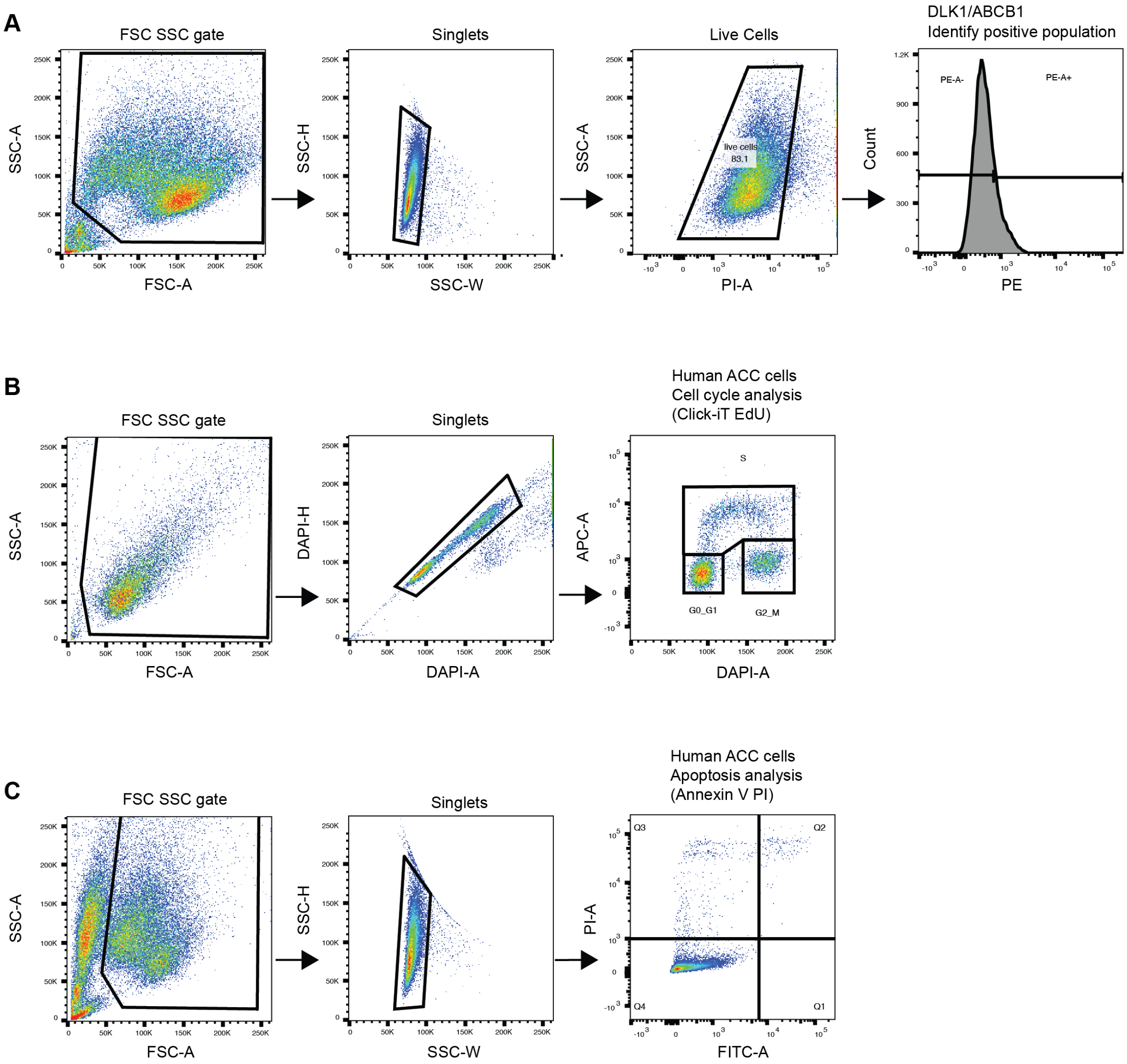

Supplementary Figure 11. Representative flow cytometric gating strategies. (A) For cell surface marker analysis (Figures 2B, 2H, 2I, 4C, 4E, 4I, 5A, 5B, 5C, 6F, 6H, and 6J, Supplementary Figures 2B, 2D, 6A, 8A, 10C, and 10G), cells were selected based on FSC/SSC profiles and then further gated for singlets and PI-negative populations. **(B)** For cell cycle analysis in Supplementary Figure 3A, cells were analyzed by APC-EdU and DAPI after single cell selection. **(C)** For apoptosis analysis in Supplementary Figure 3C, cells were analyzed by FITC-Annexin V and PI after single cell selection.
