# Supplementary material for: Identification of the Notch ligand DLK1 as an immunotherapeutic target and regulator of tumor cell plasticity and chemoresistance in adrenocortical carcinoma
Source: Nat Commun. 2025 Jul 1;16:5511. doi: 10.1038/s41467-025-60649-w (PMC12216638; doi:10.1038/s41467-025-60649-w)
Supplement: Supplementary file 2 — Description of Additional Supplementary Files [file 41467_2025_60649_MOESM2_ESM.pdf]

Description of Additional Supplementary Files

Supplementary Data 1: NCI ACC patient and tumor data

Supplementary Data 2: NCI-ACC1 PDX differential expression analysis, DESeq2. Relapsed (n=3) vs. controls (n=4). Wald test p-values and BH adjusted p-values are shown.
