# Supplementary material for: Identification of the Notch ligand DLK1 as an immunotherapeutic target and regulator of tumor cell plasticity and chemoresistance in adrenocortical carcinoma
Source: Nat Commun. 2025 Jul 1;16:5511. doi: 10.1038/s41467-025-60649-w (PMC12216638; doi:10.1038/s41467-025-60649-w)
Supplement: Supplementary file 4 — Reporting Summary [file 41467_2025_60649_MOESM4_ESM.pdf]

nature portfolio

Corresponding author(s): Nitin Roper

Last updated by author(s): Apr 15, 2025

Reporting Summary

Nature Portfolio wishes to improve the reproducibility of the work that we publish. This form provides structure for consistency and transparency in reporting. For further information on Nature Portfolio policies, see our Editorial Policies and the Editorial Policy Checklist.

Statistics

For all statistical analyses, confirm that the following items are present in the figure legend, table legend, main text, or Methods section.

n/a	Confirmed	
[ ]	[x]	The exact sample size (*n*) for each experimental group/condition, given as a discrete number and unit of measurement
[ ]	[x]	A statement on whether measurements were taken from distinct samples or whether the same sample was measured repeatedly
[ ]	[x]	The statistical test(s) used AND whether they are one- or two-sided<br>*Only common tests should be described solely by name; describe more complex techniques in the Methods section.*
[x]	[ ]	A description of all covariates tested
[x]	[ ]	A description of any assumptions or corrections, such as tests of normality and adjustment for multiple comparisons
[ ]	[x]	A full description of the statistical parameters including central tendency (e.g. means) or other basic estimates (e.g. regression coefficient) AND variation (e.g. standard deviation) or associated estimates of uncertainty (e.g. confidence intervals)
[ ]	[x]	For null hypothesis testing, the test statistic (e.g. *F*, *t*, *r*) with confidence intervals, effect sizes, degrees of freedom and *P* value noted<br>*Give P values as exact values whenever suitable.*
[x]	[ ]	For Bayesian analysis, information on the choice of priors and Markov chain Monte Carlo settings
[x]	[ ]	For hierarchical and complex designs, identification of the appropriate level for tests and full reporting of outcomes
[ ]	[x]	Estimates of effect sizes (e.g. Cohen's *d*, Pearson's *r*), indicating how they were calculated

Our web collection on statistics for biologists contains articles on many of the points above.

Software and code

Policy information about availability of computer code

Data collection	No custom code was used.
Data analysis	Cover-slipped slides were scanned with a Aperio CS-O slide scanner (Leica Biosystems).<br>FlowJo v10.8.1 software was used for flow cytometry data analysis.<br>GraphPad Prism 10.3.1 was used for graphing and statistical analysis.<br>Amnis ImageStreamX Mark II was used for imaging flow cytometry (Luminex, Austin, TX, USA).

For manuscripts utilizing custom algorithms or software that are central to the research but not yet described in published literature, software must be made available to editors and reviewers. We strongly encourage code deposition in a community repository (e.g. GitHub). See the Nature Portfolio guidelines for submitting code & software for further information.

Data

Policy information about availability of data

All manuscripts must include a data availability statement. This statement should provide the following information, where applicable:

- Accession codes, unique identifiers, or web links for publicly available datasets
- A description of any restrictions on data availability
- For clinical datasets or third party data, please ensure that the statement adheres to our policy

Previously published expression datasets re-analyzed in this study can be accessed at GSE10927 and https://portal.gdc.cancer.gov/. Data from Jain et al. was

obtained directly from the authors. Normalized RNA-seq data from the newly generated NCI ACC cohort reported in this study can be found in Supplementary Table 2 and raw RNA-seq data have been deposited in dbGaP under accession codes phs003143 and pending. Source Data are provided with this paper.

Research involving human participants, their data, or biological material

Policy information about studies with human participants or human data. See also policy information about sex, gender (identity/presentation), and sexual orientation and race, ethnicity and racism.

Reporting on sex and gender	Sex/gender are reported for all patient data in this study.
Reporting on race, ethnicity, or other socially relevant groupings	Race/ethnicity are reported for all patient data in this study.
Population characteristics	All patients were diagnosed with adrenocortical carcinoma, as confirmed by histological analysis. Detailed clinical characteristics are reported in this study.
Recruitment	ACC patient tumors used for this study were collected under NIH Institutional Review Board protocols (NCT05237934, NCT01109394, and NCT03739827).
Ethics oversight	All patients provided informed consent, and procedures were approved by the NIH Institutional Review Board according to institutional ethical guidelines.

Note that full information on the approval of the study protocol must also be provided in the manuscript.

Field-specific reporting

Please select the one below that is the best fit for your research. If you are not sure, read the appropriate sections before making your selection.

☒ Life sciences ☐ Behavioural & social sciences ☐ Ecological, evolutionary & environmental sciences

For a reference copy of the document with all sections, see nature.com/documents/nr-reporting-summary-flat.pdf

Life sciences study design

All studies must disclose on these points even when the disclosure is negative.

Sample size	Three to eight mice were used in every treatment or control arm in all mouse experiments to have sufficient statistical power to assess differences in tumor size after treatment. For in vitro studies, sample size of at least 3 was used in each experiment. Sample size and number of independent experiments are stated in the figure legends.
Data exclusions	There was no exclusions from the experiments.
Replication	To ensure reproducibility of ADCT-70 efficacy, in vitro and in vivo efficacy assays were performed using 12 ACC organoid models, 3 ACC-PDX models, 2 ACC-CDX models, 3 SCLC xenograft models, 3 ACC cell lines, 1 PCPG cell line, and 3 SCLC cell lines.For in vitro cell line data, we performed at least 3 biologically independent experiments for all key data presented and all attempts at replication were successful. For in vitro organoid data, we performed either 1 or 2 biologically independent experiments depending on model availability.
Randomization	For in vivo mouse tumor studies, mice were randomly assigned to each treatment group after tumor reached a certain size (~100-200 mm3).
Blinding	Investigators were not blinded to group allocation duration data collection and/or analysis as it was not technically feasible. All the data analysis is based on absolute quantification by instrumentation and does not include subjective factors.

Reporting for specific materials, systems and methods

We require information from authors about some types of materials, experimental systems and methods used in many studies. Here, indicate whether each material, system or method listed is relevant to your study. If you are not sure if a list item applies to your research, read the appropriate section before selecting a response.

Materials & experimental systems

n/a	Involved in the study	
[ ]	[x]	Antibodies
[ ]	[x]	Eukaryotic cell lines
[x]	[ ]	Palaeontology and archaeology
[ ]	[x]	Animals and other organisms
[ ]	[x]	Clinical data
[x]	[ ]	Dual use research of concern
[x]	[ ]	Plants

Methods

n/a	Involved in the study	
[x]	[ ]	ChIP-seq
[ ]	[x]	Flow cytometry
[x]	[ ]	MRI-based neuroimaging

Antibodies

Antibodies used

IHC analysis:
DLK1 (Abcam, Cat# ab21682, 1:2000), SYP (Roche Diagnostics, Cat# 790-4407, clone SP11; pre-diluted), SF1 (Perseus Proteomics, Cat# PP-N1665-00, clone N1665, 1:200), Ki67 (Agilent Technologies, Cat# M7240, clone MIB-1, 1:200)

Flow cytometry analysis:
anti-human DLK1 primary antibody (AdipoGen Life Sciences, Cat# AG-20A-0070-C100, PF299-1, 1:100 per one million cells), PE-conjugated Goat anti-mouse IgG (H+L) cross-adsorbed secondary antibody (Invitrogen, Cat# P-852, 1:500), human DLL3 PE-conjugated antibody (R&D Systems, Cat# FAB4315P, 10 μL per one million cells), Goat IgG PE-conjugated isotype control antibody (R&D Systems, Cat# IC108P, 10 μL per one million cells), PE anti-human CD243 (MDR-1) antibody (BioLegend, Cat# 348606, clone UIC2, 5 μl per one million cells in 100 μl wash buffer), PE mouse IgG2a, κ isotype control (FC) antibody (BioLegend, Cat# 400214, clone MOPC-173, 5 μl per one million cells in 100 μl wash buffer)

Imaging flow cytometry analysis:
human Pref-1/DLK1/FA1 APC-conjugated antibody (R&D Systems, Cat# FAB1144A, 10 μl per one million cells for 1 hour in 2 ml of cell culture media at 37°C), mouse IgG2B APC-conjugated isotype control antibody (R&D Systems, Cat# IC0041A, 10 μl per one million cells for 1 hour in 2 ml of cell culture media at 37°C)

Western blot analysis:
rabbit anti-DLK1 antibody (Cell Signaling Technology, Cat# 2069, 1:1000), mouse anti-phospho-histone H2A.X (Ser139) antibody (Millipore, Cat# 05-636, 1:1000), rabbit anti-cleaved caspase-3 (Asp175) antibody (Cell Signaling Technology, Cat# 9661, 1:1000), rabbit-cleaved PARP (Asp214) antibody (Cell Signaling Technology, Cat# 9541, 1:1000), rabbit anti-Notch1 (D1E11) XP® antibody (Cell Signaling Technology, Cat# 3608, 1:1000), rabbit anti-cleaved Notch1 (Val1744) (D3B8) antibody (Cell Signaling Technology, Cat# 4147, 1:1000), rabbit anti-Synaptophysin (D8F6H) XP® (Cell Signaling Technology, Cat# 36406, 1:1000), rabbit anti-CYP17A1 antibody (Cell Signaling Technology, Cat# 17334, 1:1000), mouse anti-α-Tubulin antibody (Sigma-Aldrich, Cat# T9026, 1:1500), donkey anti-rabbit IgG-HRP (Cytiva, Cat# NA934, 1:5000), sheep anti-mouse IgG-HRP (Cytiva, Cat# NA931, 1:5000)

Validation

All antibodies used in this study were certified by the producing companies with respect to their specificity and quality.

Eukaryotic cell lines

Policy information about cell lines and Sex and Gender in Research

Cell line source(s)

Human ACC cell lines CU-ACC1 and CU-ACC2 were obtained from the University of Colorado School of Medicine, Aurora, CO, USA. Human ACC cell line H295R (CRL-2128) was obtained from The American Type Culture Collection (ATCC). Human PCPG cell line hPheo1 was a gift from Dr. Karel Pacak at the Eunice Kennedy Shriver National Institute of Child Health and Human Development (NICHD), NIH, Rockville, MD, USA. Human SCLC cell lines H146 (HTB-173) and H524 (CRL-5831) were obtained from ATCC. Human SCLC cell line H1436 was obtained from Dr. Haobin Chen at Washington University School of Medicine, St. Louis, MO, USA. The patient-derived xenografts used in this study, including 164165 and 592788 were obtained from the NCI Patient-Derived Models Repository (PDMR) within the NCI Developmental Therapeutics Program (Bethesda, MD, USA). POBNCI_ACC004 PDX was developed by the NCI Pediatric Oncology Branch, Bethesda, MD, USA.

Authentication

Short tandem repeat DNA profiling was used for cell line authentication.

Mycoplasma contamination

All cell lines were Mycoplasma negative.

Commonly misidentified lines
(See ICLAC register)

No commonly misidentified cell lines were used in the study.

Animals and other research organisms

Policy information about studies involving animals; ARRIVE guidelines recommended for reporting animal research, and Sex and Gender in Research

Laboratory animals

Seven-week-old female NOD/SCID/IL-2Rgcnull (NSG) mice and female (Crl:NU(NCr)-Foxn1nu) athymic nude mice were used in this study. Both male and female NSG mice were used for PDX passaging. Mice were housed in IVM Microisolator conditions designed for

	immune compromised mice at 22 degrees Celsius with humidity of 50% and a 12 hour light dark cycle (6 am - 6 pm).
Wild animals	No wild animal were involved in our study.
Reporting on sex	ACC is more common in females than males. Only female mice were used in this study to match sex of patient from tumor was derived.
Field-collected samples	This study did not involve field collected samples.
Ethics oversight	All animal procedures reported in this study were approved by the NCI Animal Care and Use Committee (ACUC) and in accordance with federal regulatory requirements and standards, protocol DTB-007.

Note that full information on the approval of the study protocol must also be provided in the manuscript.

Clinical data

Policy information about clinical studies

All manuscripts should comply with the ICMJEguidelines for publication of clinical research and a completedCONSORT checklist must be included with all submissions.

Clinical trial registration	NCT05237934, NCT01109394, and NCT03739827
Study protocol	Full clinical protocols for these studies are available upon request.
Data collection	ACC patients were recruited based on necessity for surgical tumor resection. Geographical bias may skew the generalizablility of our patient population. Population characteristics of the human research participants are included in Supplementary Data 1.
Outcomes	Ongoing

Plants

Seed stocks	N/A
Novel plant genotypes	N/A
Authentication	N/A

Flow Cytometry

Plots

Confirm that:

- [x] The axis labels state the marker and fluorochrome used (e.g. CD4-FITC).
- [x] The axis scales are clearly visible. Include numbers along axes only for bottom left plot of group (a 'group' is an analysis of identical markers).
- [x] All plots are contour plots with outliers or pseudocolor plots.
- [x] A numerical value for number of cells or percentage (with statistics) is provided.

Methodology

Sample preparation	Adherent CU-ACC1, CU-ACC2, H295R ACC cells and hPheo1 PCPG cells were harvested by Trypsin-EDTA. Suspension H524, H146, and H1436 SCLC cells were harvested by centrifugation. For organoid models, cells embedded in Matrigel matrix were recovered by Dispase. Single cells from suspension SCLC cells or organoids were prepared after Trypsin-EDTA and filtration through 70µm cell strainer. For ACC patient tumor and xenograft models, tumors were minced into tiny fragments and digested with collagenase type 4 and DNase I. Tumor fragments were filtered through 70µm cell strainer. All samples were stained in FACS buffer (PBS containing 1% BSA and 0.1% sodium azide) with different antibody dilutions. For image flow cytometry analysis, ACC cell lines were incubated with APC-conjugated DLK1 antibody or isotype control antibody for 1 hour at 37°C. Cell monolayer was then collected and rinsed with cold PBS twice and resuspended. For cell cycle analysis, cells treated with ADCT-701 or B12-PL1601 were labeled with Click-iT™ EdU in a 37°C for 1 hr. Cells were then fixed and permeabilized. Click-iT™ Plus reaction cocktail was added in cells. Cells were then stained with DAPI for DNA content. For apoptosis analysis, cells treated with ADCT-701 or B12-PL1601 were were washed with cold FACS buffer and resuspended in Annexin V binding buffer and stained with FITC-Annexin V and PI.

Instrument	BD LSRFortessa™ (BD Bioscience) and Amnis ImageStreamX Mark II imaging flow cytometry (Luminex, Austin, TX, USA)
Software	BD FACS DIVA software v8.0.1 was used for data acquisition and FlowJo v10.8.1 software was used for data analysis.
Cell population abundance	The study did not include cell sorting.
Gating strategy	For cell surface marker analysis, cell debris and cell doublets were eliminated in FSC/SSC plots, and then alive cells were gated as PI negative cells. Only live cells were analyzed for cell surface binding of antibodies. For cell cycle analysis, cell debris and doublets were removed from analysis using FSC/SSC and DAPI-A/DAPI-H gates. Cells were then analyzed for APC-EdU/DAPI. For apoptosis analysis, cell debris and cell doublets were eliminated in FSC/SSC plots, and then cells were analyzed for FITC-Annexin V/PI plot.

☒ Tick this box to confirm that a figure exemplifying the gating strategy is provided in the Supplementary Information.
